# Identification of CPT2 as a prognostic biomarker by integrating the metabolism-associated gene signature in colorectal cancer

**DOI:** 10.1186/s12885-022-10126-0

**Published:** 2022-10-04

**Authors:** Jiaxin Liu, Yimin Li, Qing Xiao, Yuanyuan Li, Yuqian Peng, Yaqi Gan, Guang Shu, Hanxi Yi, Gang Yin

**Affiliations:** 1 Department of Pathology, Xiangya Hospital, School of Basic Medical Sciences, Central South University, Changsha, 410000 China; 2 School of Basic Medical Sciences, Central South University, Changsha, Hunan Province China; 3 China-Africa Research Center of Infectious Diseases, School of Basic Medical Sciences, Central South University, Changsha, Hunan Province China; 4 National Clinical Research Center for Geriatric Disorders, Xiangya Hospital, Central South University, Changsha, Hunan China

**Keywords:** CPT2, Colorectal cancer, Metabolism, TCGA, GEO

## Abstract

**Background:**

The incidence of colorectal cancer (CRC) is considered to be the third-highest malignant tumor among all carcinomas. The alterations in cellular bioenergetics (metabolic reprogramming) are associated with several malignant phenotypes in CRC, such as tumor cell proliferation, invasion, metastasis, chemotherapy resistance, as well as promotes its immune escape. However, the expression pattern of metabolism-associated genes that mediate metabolic reprogramming in CRC remains unknown.

**Methods:**

In this study, we screened out CPT2 by investigating the function of a series of metabolism-related genes in CRC progression by integrating the data from the TCGA and GEO databases. Next, we collected CRC tissues (*n* = 24) and adjacent non-tumor tissues (*n* = 8) and analyzed mRNA levels by qRT-PCR, and proteins levels of CPT2 in CRC cell lines by western blotting. CCK-8 assay, colony formation assay, Edu assay and flow cytometry assay were performed to assess the effects of CPT2 on proliferation in vitro.

**Results:**

We identified 236 metabolism-related genes that are differentially expressed in colorectal cancer, of which 49 up-regulated and 187 down-regulated, and found CPT2 as the most significant gene associated with favorable prognosis in CRC. It was revealed that CPT2 expression was consistently down-regulated in CRC cell lines and tissues. Moreover, knockdown of CPT2 could promote the proliferative ability of CRC cells, whereas over-expression of CPT2 significantly suppressed the cell growth.

**Conclusion:**

In summary, CPT2 can provide new insights about the progression and occurrence of the tumor as it acts as an independent prognostic factor in CRC sufferers.

**Supplementary Information:**

The online version contains supplementary material available at 10.1186/s12885-022-10126-0.

## Introduction

Colorectal cancer (CRC) is known to be the third most universally diagnosed cancer, and the second most common cause of cancer death [1]. The CRC formation and growth is a long-term evolutionary process, and starts from benign adenomatous polyps, after that it turns into an advanced adenoma alongside high-grade dysplasia and finally into an invading carcinoma [2]. In recent years, the mortality rate of CRC has been reduced due to the development of new and effective anti-cancer drugs. However, due to the higher incidence of local recurrence and distant metastasis, the long-term survival rate of CRC patients is still not ideal [3, 4]. Therefore, the identification of new molecular biomarkers is urgently needed to improve the therapeutic effect of currently used drugs against CRC as well as to explore new therapeutic strategies.

In cancer cells, metabolic reprogramming is most commonly occurring and is a trait of tumor cells [5, 6]. It has also been observed that in CRC, metabolomic alterations can partially occur. In the occurrence and development of CRC, specific metabolic pathways may interfere [7]. TCA cycle and amino acid turnover are deregulated by CRC cells [8]. Abnormal metabolic pathways, such as impaired tricarboxylic acid cycle, decreased gluconeogenesis, and inhibition of glucuronic acid synthesis may play an important part in the CRC pathogenesis [9]. It is worth noticing that some of the CRC genetic drivers are identified as cancer metabolic regulators such as Wnt, KRas, and p53 [10–12]. The expression pattern of metabolism-associated genes that mediate metabolic reprogramming in CRC remains unknown. The current study aims to find out various aspects of metabolism-related genes in CRC. To explore their prognostic value in CRC, the hub genes were screened integrating the data from the TCGA-CRC and GEO databases. Through Cox regression and survival prognostic analysis, it was identified that the down-regulated expression of Carnitine palmitoyl transferase 2 (CPT2) is related to poor prognosis of CRC.

CPT2, which is located on the inner surface of the inner membrane of mitochondria, plays a significant role in catalyzing the conversion of acyl groups from acylcarnitine to acyl-CoA [13]. Studies have revealed that in cancer metabolism, fatty acid oxidation (FAO) plays a vital role in the homeostasis of cell energy [14]. CPT2 is a key regulatory enzyme of FAO, which is closely related to the invasion, proliferation, migration, and chemical resistance to cisplatin of hepatoma cells [15]. CPT2 down-regulation is important in enabling hepatocellular carcinoma (HCC) cells to escape lipotoxicity but also hepatocarcinogenesis is enhanced [16]. It is worth noting that enhanced expression of CPT2 was not only found in the recurrence of human breast cancer but was also found involved in the poor chances of breast cancer recovery [17]. However, its function is still unclear in CRC. The current research revealed that CPT2 impeded the ability of proliferation, thus offering a new target for improving the therapeutic effect of drugs against CRC.

## Materials and methods

### Data collection

The TCGA cohort data were available at the Genomic Data Commons (GDC) website (https://portal.gdc.cancer.gov/). Two independent cohorts of CRC data were downloaded from the GEO database (http://www.ncbi.nlm.nih.gov/geo/): GSE44076 [18] and GSE21510 [19]. Then, data were analyzed by using R-studio software. Distinctive expression of metabolism-related genes was noticed by using the “edgeR package” of |log2FC| > 1 and FDR < 0.05 as criteria for screening the optimal characterized genes. The distinctive expression between normal and cancer samples were shown by the heatmap and volcano plot.

### PPI network and module analysis

For the construction of the PPI network of genes which are differentially expressed, Cytoscape software and Search Tool for the Retrieval of Interacting Genes/Proteins (STRING) were used [20]. The MCODE (Molecular Complex Detection) app determined the top three modules. For identified genes, KEGG pathway enrichment analysis was carried by employing the Database for Annotation, Visualization, and Integrated Discovery (DAVID).

### Gene set enrichment analysis (GSEA)

GSEA was used to identify potential pathways of the gene signature and to find the enriched terms in Hallmark gene sets in the TCGA-CRC cohort. The statistically significant values were *p* < 0.05 and p adjust < 0.05 and the top 5 pathways were performed.

### Patients and samples

Xiangya Hospital of Central South University (Changsha, China) was approached to obtain colorectal cancer (CRC) specimens and adjacent normal tissues. A total of 24 freshly resected cancer tissue samples and 8 adjacent noncancerous colonic tissue samples from patients with CRC diagnosed between 2011 and 2013 were used in this study. The informed consent was taken from all the sufferers and was informed about the research. The current study was conducted after the approval of the Ethics Committee of Xiangya Hospital in agreement with the guidelines set forth by the Declaration of Helsinki.

### Cell lines and cell culture

American Type Culture Collection (ATCC; http://www.atcc.org/) was approached for the purchasing of normal cell line NCM460 and the CRC cell lines (SW480, HCT116, and SW620). Professor Wancai Yang (Institute of Precision Medicine, Jining Medical University) humbly donated LOVO cell lines. RPMI-1640 medium was used to maintain all cell lines alongside 10% FBS (fetal bovine serum) (Biological Industries, Kibbutz belt haemek, Israel). Under the temperature of 37 °C and 5% CO_2_, all cells were cultured and incubated.

### Cell transfection

For overexpression of CPT2 in CRC cells, the full-length CPT2 cDNA was amplified from HCT116 cell and then inserted into pcDNA3.1 vector. RiboBio (Guangzhou, China) was approached to purchase Small interfering RNAs (siRNAs) targeting CPT2 for the purpose to silence CPT2. The following was the sequence of siRNAs targeting CPT2: CPT2 si#1: GTAGCACTGCCGCATTCAA, CPT2 si#2: GACCCTGGTTTGATATGTA. Small interfering RNAs (siRNA) transfection was carried out from the jetPRIME DNA & siRNA Transfection Reagent (PolyPlus-transfection, France). The cells were collected after 48 hours of transfection and western blot analysis was used to confirm the knockdown efficacy of the cells and was used for subsequent tests.

### Cell proliferation assay

The ability of cell proliferation was detected by Cell Counting Kit-8 (CCK8) assay, Colony formation assay and EDU assay. After seeding 3 × 10^3^ cell suspension in 96-well plates, the cells were treated with CCK-8 (10 μl/well) for 2 h, and the optical density value (OD value) was detected at 450 nm.

For colony formation assay, 6-well plates were used in which 500 infected HCT116 and SW480 cells were placed. The surviving cell colonies were fixed after 10 days of incubation and were then stained with crystal violet and the numbers of colonies were estimated using the ImageJ.

The EdU (RiboBio, Guangzhou, China) assay was used in accordance with the described standard protocol. The results were calculated with ImageJ. All experiments were performed repeatedly at least three times.

### Extraction of RNA and quantitative reverse transcriptase polymerase chain reaction (qRT-PCR)

The extraction of the total RNA was carried out by using Trizol reagent (Vazyme, Nanjing, China). Go Script Reverse Transcription System (Promega, Madison, WI, USA), was used for perform reverse transcription PCR. On an ABI Prism 700 thermal cycler (Applied Biosystems, Foster City, CA, USA), real-time qPCR with GoTaq qPCR Master Mix (Promega, Madison, WI, USA) was performed. The following are primer sequences: CPT2 (forward primer: TCCTGTCCACGAGCACACTGAG; reserve primer: AGCATACCCAACACCAAAGCCATC); GAPDH (forward primer: CTGGGCTACACTGAGCACC; reserve primer: AAGTGGTCGTTGAGGGCAATG). For the purpose of internal control, GAPDH was employed.

### Western bolt

The extraction of the total protein was carried out from the targeted cells by employing RIPA buffer which contains protease inhibitors and BCA protein assay was used to measure the protein concentrations of supernatants. SDS-PAGE was carried out with a total of 30 μg protein. The earlier researches also highlighted the procedure [21]. Anti-CPT2 (1:1000, ab181114, Abcam, UK) and anti-GAPDH (1:5000, 60,004–1-Ig, proteintech, China) were used as primary antibodies. The secondary antibodies used in this study were as follows: Anti-Mouse IgG(H + L) (1:5000, SA00001–1, proteintech, China) and Anti-Rabbit IgG (H + L) (1:5000, SA00001–2, proteintech, China).

### Cell cycle analysis

After 48 hours of interference with CPT2 siRNA, HCT116 cells were harvested, then centrifuged and resuspended in 1x PBS. The cells were fixed in 70% ethanol overnight. The next day, after washing with PBS solution and centrifuging, the cells were stained with PI (propidium iodide) and analyzed by the FACSCalibur system (BD Biosciences, San Jose, California, USA).

### Statistical analysis

Statistical analysis was carried out by using R Studio (R version 3.6.3), Graphpad Prism Software 8.0, and SPSS version 20.0. Data are shown as means ± standard deviation (SD). For the analysis of statistical significance between groups, Student’s t-test was used. The significance of statistics was set at *p* < 0.05.

## Results

### Screening of differentially expressed genes (DEGs) in CRC

**Fig. 1:**
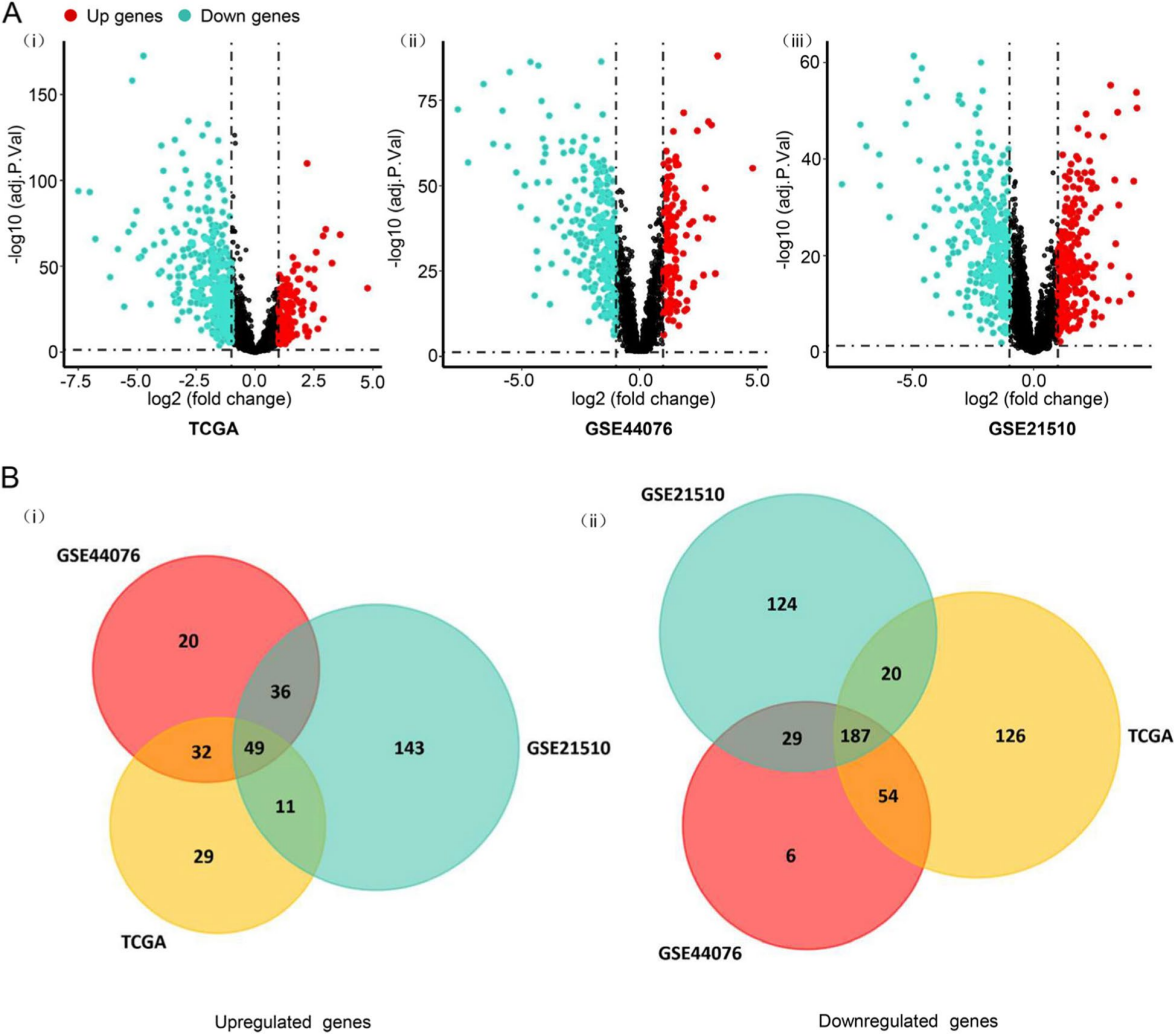
Differentially expressed genes and common differentially expressed genes in three datasets. **A** The volcano plot represents the differentially expressed genes in (i) TCGA-CRC, (ii) GSE44076, and (iii) GSE21510, accordingly. The red nodes indicate upregulated genes. The blue nodes represent downregulated genes. **B** Commonly upregulated genes (i) and downregulated genes (ii) in three datasets, the various colored areas show different data sets

To find out the role of metabolic genes in CRC, 2761 metabolism-related genes were obtained from the study conducted by Zeribe Chike Nwosu [22] and Richard Possemato [23]. Gene expression profiling was collected from three chips which are TCGA-CRC, GSE44076, and GSE21510. The gene expression profiling was analyzed by the “limma” package of R software [24]. All the differentially expressed genes were screened out in three expression profiles by using FDR < 0.05 and |log2FC| > 1 as the threshold. The DEGs of the three datasets were indicated as volcano plots and heat map in Fig. 1A and Supplementary Fig. 1A. After being overlapped, the common 49 up-regulated and 187 down-regulated genes were observed (Fig. 1B).

### Key module and hub genes screening through bioinformatics analyses

**Fig. 2:**
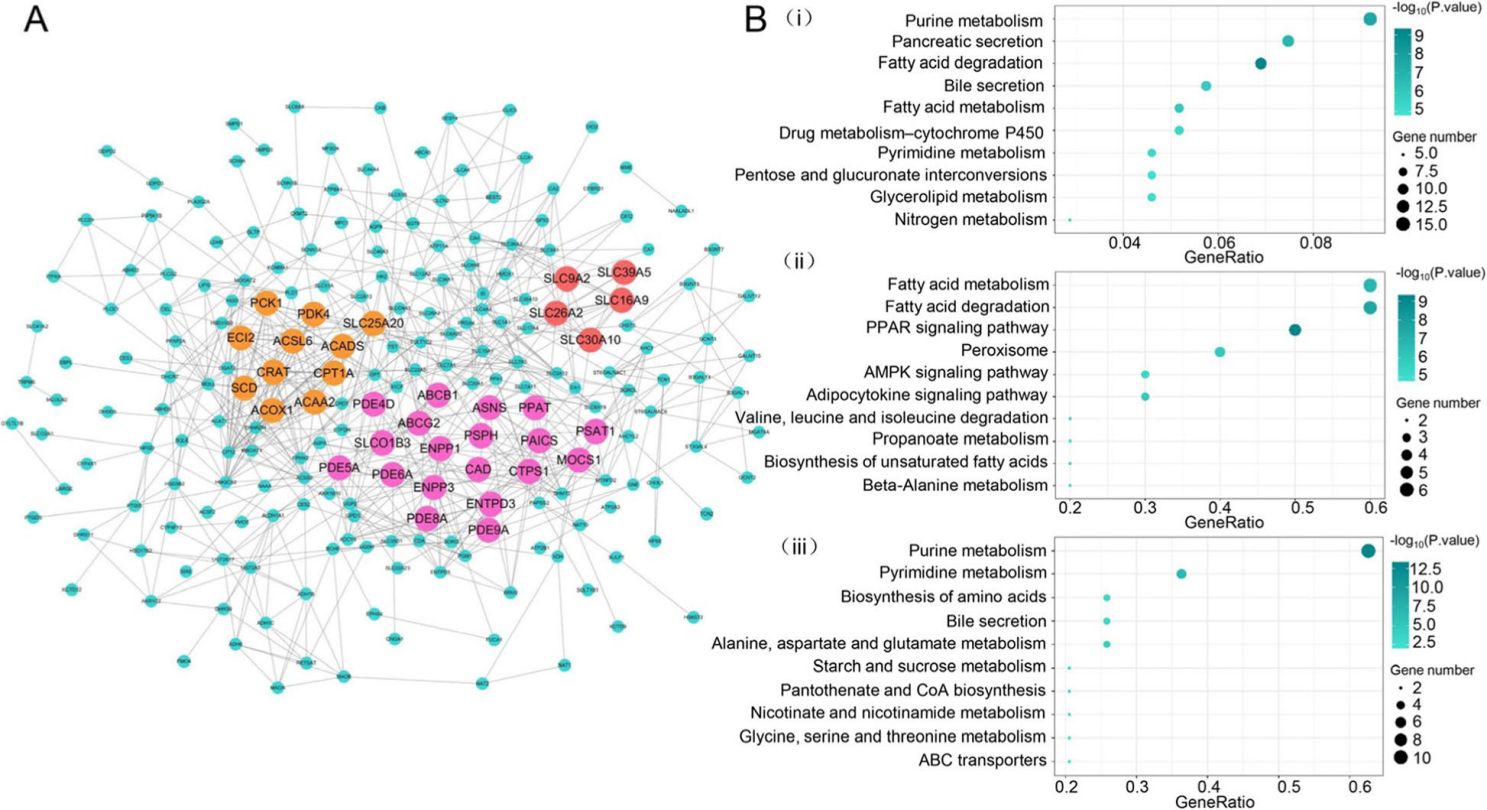
PPI network analysis of differentially expressed genes. **A** The differentially expressed genes were imported into STRING to construct PPI networks. The top three modules were identified from the PPI network. **B** analysis of gene enrichment of the KEGG pathway for individual module 1(i), module 2(ii), module 3(iii)

The STRING database and Cytoscape software were used to create a PPI network to further find out the biological roles of these DEGs. Three significant modules were taken out from the PPI network containing 236 genes by MCODE (Fig. 2A). To explore potential biological processes associated with CRC, KEGG pathway enrichment was then carried out for the above three modules. Figure 2B showed the result of KEGG pathway enrichment. Purine metabolism, pancreatic secretion, and fatty acid degradation are the most significant pathway of module 1. The genes of module 2 are enriched in fatty acid metabolism, fatty acid degradation, and the PPAR signaling pathway. The genes of module 3 are significantly enriched in purine metabolism, pyrimidine metabolism, and biosynthesis of amino acids.

The top ten hub genes that have enhanced integration were explored to ensure the key metabolism-related genes involved in the CRC development, which comprises CPT2, ACOX1, EHHADH, HMGCS2, ENPP1, ECI2, ACSL6, SLC25A20, DGAT2, PCK1(Table 1).

**Table 1:** The PPI network of top ten hub genes

Symbol	Degree	Classification	LogFC		
			GSE21510	GSE44076	TCGA
CPT2	21	Fatty Acid	−1.64	−1.36	− 1.41
ACOX1	18	Fatty Acid	−1.5	−1.47	− 1.44
EHHADH	16	Fatty Acid	−1.05	−1.21	− 1.18
HMGCS2	16	Mevalonate	−2.8	−3.13	− 3.28
ENPP1	16	Nucleotide	−1.06	− 1.21	− 1.21
ECI2	16	Fatty Acid	−1.5	−1.3	− 1.58
ACSL6	16	Fatty Acid	3.92	2.48	2.14
SLC25A20	16	Small Molecule Transport	−2.02	−1.37	−1.49
DGAT2	16	Fatty Acid	1.14	1.21	1.75
PCK1	16	Carbohydrate Storage	−2.75	−3.65	−3.34

### The correlation between CPT2 expression and CRC clinicopathological features

**Fig. 3:**
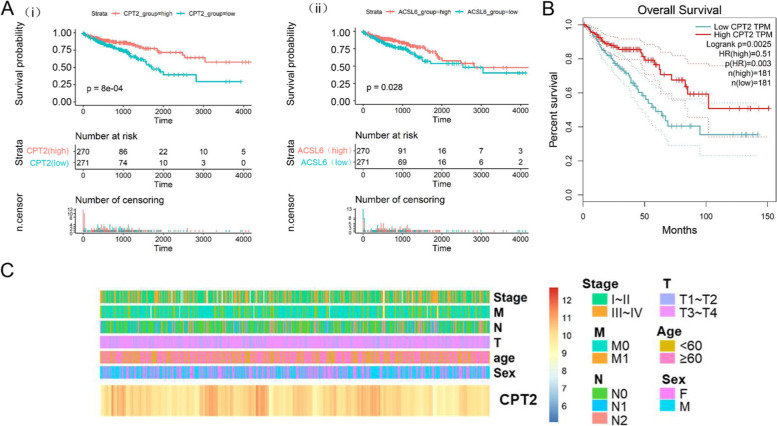
The correlation of CPT2 expression with CRC clinicopathological features. **A** The Kaplan-Meier survival curve for patients in the low- and high- risk groups of CPT2 (i) and ASCL6 (ii) in the TCGA-CRC cohort. **B** Kaplan-Meier curves for CPT2 in CRC cohort in GEPIA. **C** The distribution of clinicopathological characteristics of CPT2

Further analysis of the top ten hub genes in the TCGA-CRC cohort was carried out to indicate their clinical significance and potential function in CRC while considering their significant association of metabolism-related genes with the development of CRC. Tumor samples within the TCGA-CRC dataset were divided into two groups, which are named as high-risk group and low-risk group, according to the median expression value to plot the Kaplan-Meier survival curves. The results indicated that only CPT2 and ASCL6 significantly affected the overall survival of CRC patients. It was shown by the Kaplan-Meier curve that the overall survival time of patients in the low-risk group was notably extended as compared to those in higher ones (Fig. 3A). Then CPT2 and ASCL6 were further validated by the GEPIA database. Only the overall survival analysis of CPT2 showed statistical differences (*P* < 0.01) (Fig. 3B). The distribution of clinicopathological features of CPT2 was analyzed in CRC (Fig. 3C). Table 2 indicates that the lower expression of CPT2 was significantly relevant to the lymph node metastasis (*P* = 0.001), tumor grade (*P* = 0.007), and TNM stage (*P* = 0.001), whereas no significant relevance was noticed with age, gender, or distant metastasis.

**Table 2:** Correlations between CPT2 expression and clinicopathologic features in 541 colorectal cancer patients from TCGA cohorts

Characteristics	CPT2		*P* Value
	Low	High	
Gender			0.246
Male	151	137	
Female	120	133	
Age (years)			0.376
< 60	72	81	
≥ 60	199	189	
Tumor grade			**0.007**
T1 ~ T2	42	67	
T3 ~ T4	229	203	
Lymph node metastasis			**0.001**
N0	140	179	
N1 ~ N2	131	91	
Distant metastasis			0.382
M0	198	206	
M1	71	62	
TNM Stage			**0.001**
I ~ II	133	166	
III ~ IV	133	94	

Bold type represents statistical significance

**Fig. 4:**
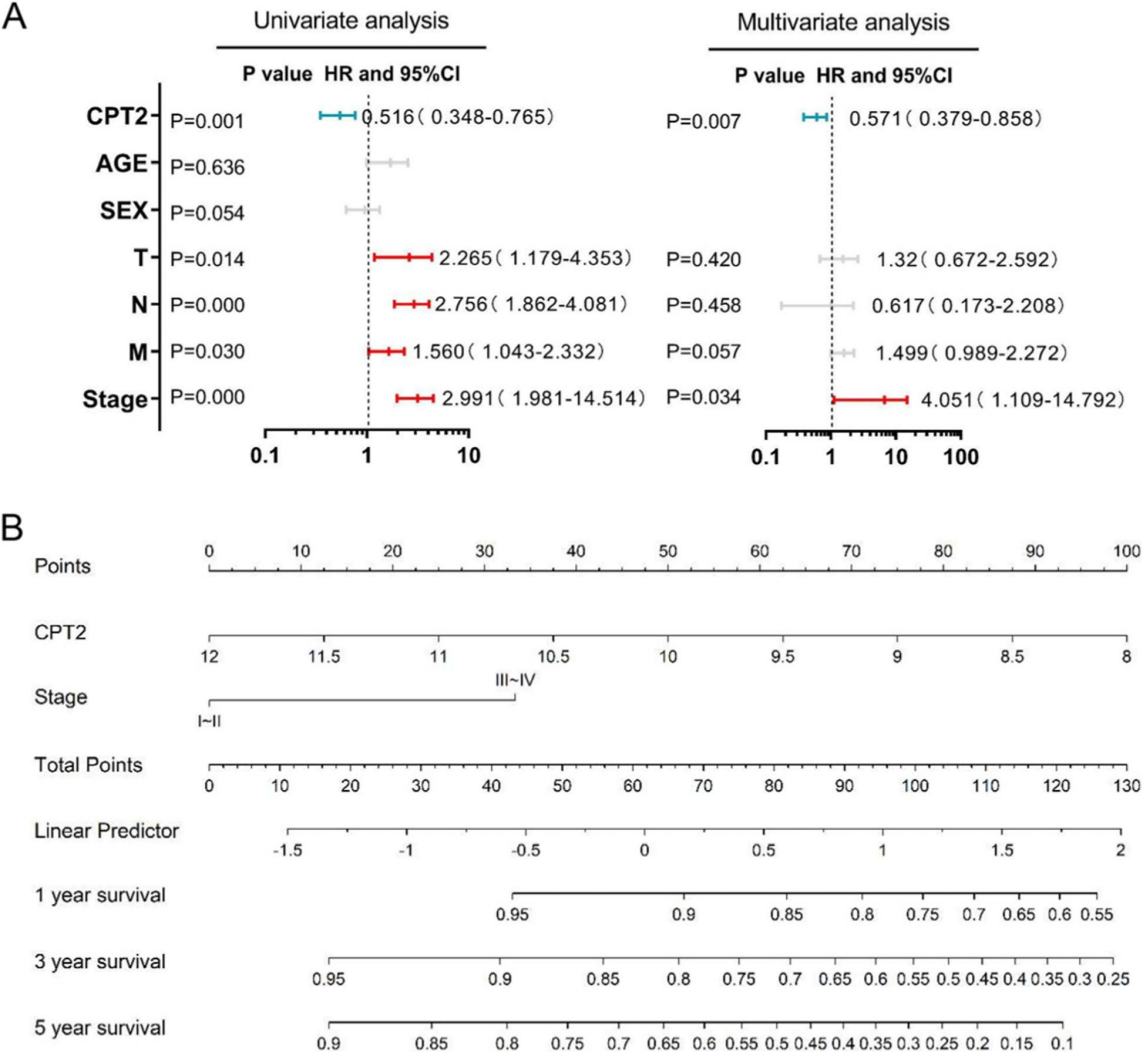
The correlation of CPT2 expression with CRC overall survival features. **A** Univariate and multivariate analyses for predictors of overall survival in the TCGA-CRC dataset. **B** The nomogram plot was built based on the expression of CPT2 and stage in CRC. Then directly convert total points to particular 1-year, 3–year, and 5–year related survival probabilities

Univariate and multivariate Cox regression analyses were carried out for the data from the TCGA-CRC dataset in order to find out the correlation of CPT2 expression with CRC survival (Fig. 4A). The results of univariate and multivariate Cox regression analysis explored that both TNM stage and CPT2 expression acted as an independent prognostic factor for overall survival (OS) (Fig. 4A). Therefore, a nomogram was constructed to predict the survival probability of CRC patients which integrates with the significant prognostic factors (Fig. 4B). By adding up the total score and locating it on the total point scale, the total points were directly converted into particular 1-year, 3-year, and 5-year related chances of survival.

### Validation of the gene expression levels of CPT2 in the public database and our CRC tissues and cells

**Fig. 5:**
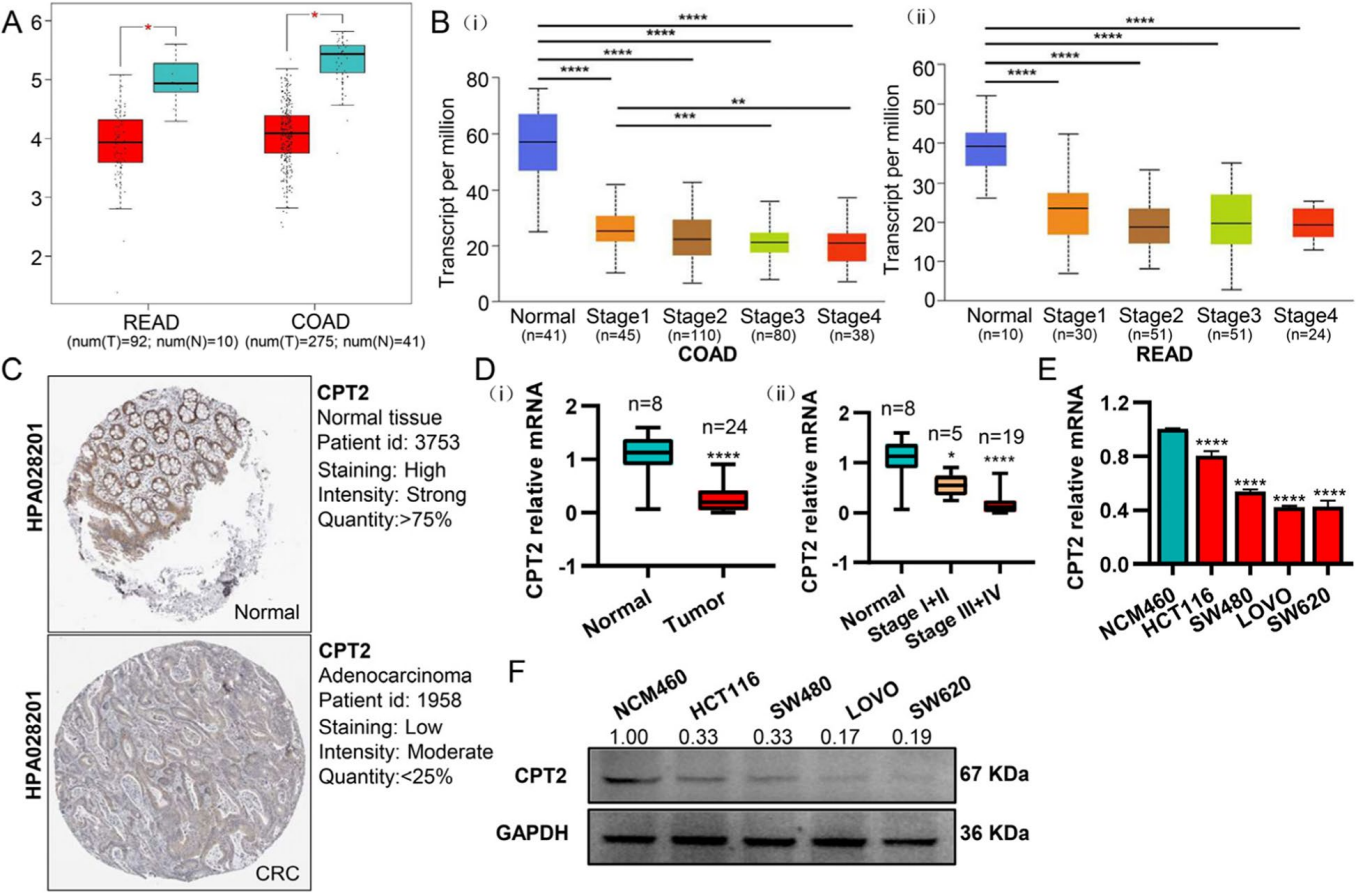
CPT2 is down-regulated in colorectal cancer. **A** Boxplot showing the correlation of CPT2 expression in normal and READ (left) or COAD (right) samples. **B** Boxplot showing the correlation between CPT2 expression and tumor stage in COAD (i) and READ (ii) patients in the UALCAN database. **C** The representative protein expression of CPT2 in CRC and normal tissue from the Human Protein Atlas (HPA) dataset. **D** The expression of CPT2 was analyzed by qRT-PCR in CRC (*n* = 8) and normal colorectal tissues (*n* = 24) (i), (ii) showing the correlation between CPT2 expression and tumor stage in patients. **E**-**F** The expression of CPT2 was analyzed by qRT-PCR (E) and Western blotting (F) in NCM460 and CRC cell lines. Data are presented as mean ± SD from three independent experiments. *P < 0.05, **P < 0.01, ***P < 0.001, ****P < 0.0001

The expression of CPT2 in COAD and READ was further explored through the GEPIA website and the results showed that by comparing with normal tissues, the level of CPT2 expression was extremely low in tumor tissues (Fig. 5A). In the UALCAN database, it was observed that CPT2 was down-regulated in CRC tissues and the expressions of CPT2 were significantly negatively associated with tumor stage (Fig. 5B). The representative protein expression of CPT2 was explored in the Human Protein Atlas (www.proteinatlas.org). The current research explored that CPT2 had a strong expression in the normal tissues and moderate expression in the CRC tissues (Fig. 5C).

We further validated CPT2 expression levels in CRC tissues and cells. To detect the level of expression in CRC tissues, qRT-PCR was performed and expression of CPT2 in CRC tissues was noticed, which indicated that the level of expression of CPT2 was particularly lower in CRC tissues as compared to normal tissues (Fig. 5D i). We also found the expression of CPT2 decreased in tumor stage III-IV compared to stage I-II (Fig. 5D ii). The expression level of CPT2 was further assessed in non-cancerous NCM460 colon cells and CRC cell lines LOVO, SW480, SW620, and HCT116. It was noticed from the results of the study that in tumor cell lines, both the protein and mRNA levels of CPT2 was significantly down-regulated (Fig. 5E, F), which was the same as the results of the public database.

### Down-regulated CPT2 promotes cell proliferation in CRC

To find out the functions and possible pathways of CPT2, gene set enrichment analysis (GSEA) was employed. To determine the enrichment score of potential pathways in the gene sets positively or negatively correlated with CPT2 expression, we performed GSEA in the TCGA-CRC dataset. The Hallmark gene sets used for the enrichment analysis were downloaded from the Molecular Signatures Database (http://software.broadinstitute.org/gsea/index.jsp). Briefly, we firstly generated an ordered genes list according to their correlation with CPT2 expression, then GSEA was performed by using R. The statistically significant values were *p* < 0.05 and p adjust < 0.05, and the top 5 pathways were performed. It was identified by GSEA that CPT2 was negatively enriched in E2F targets, epithelial-mesenchymal transition, G2M checkpoint, MYC targets V1, and MYC targets V2, whereas having positive relation with adipogenesis, fatty acid metabolism, heme metabolism, oxidative phosphorylation, and xenobiotic metabolism processes (Supplementary Fig. 2A). In earlier researches, the function of CPT2 in the progression of liver cancer formation was observed [16], and GSEA shows that CPT2 is negatively correlated with the G2M checkpoint in CRC.

**Fig. 6:**
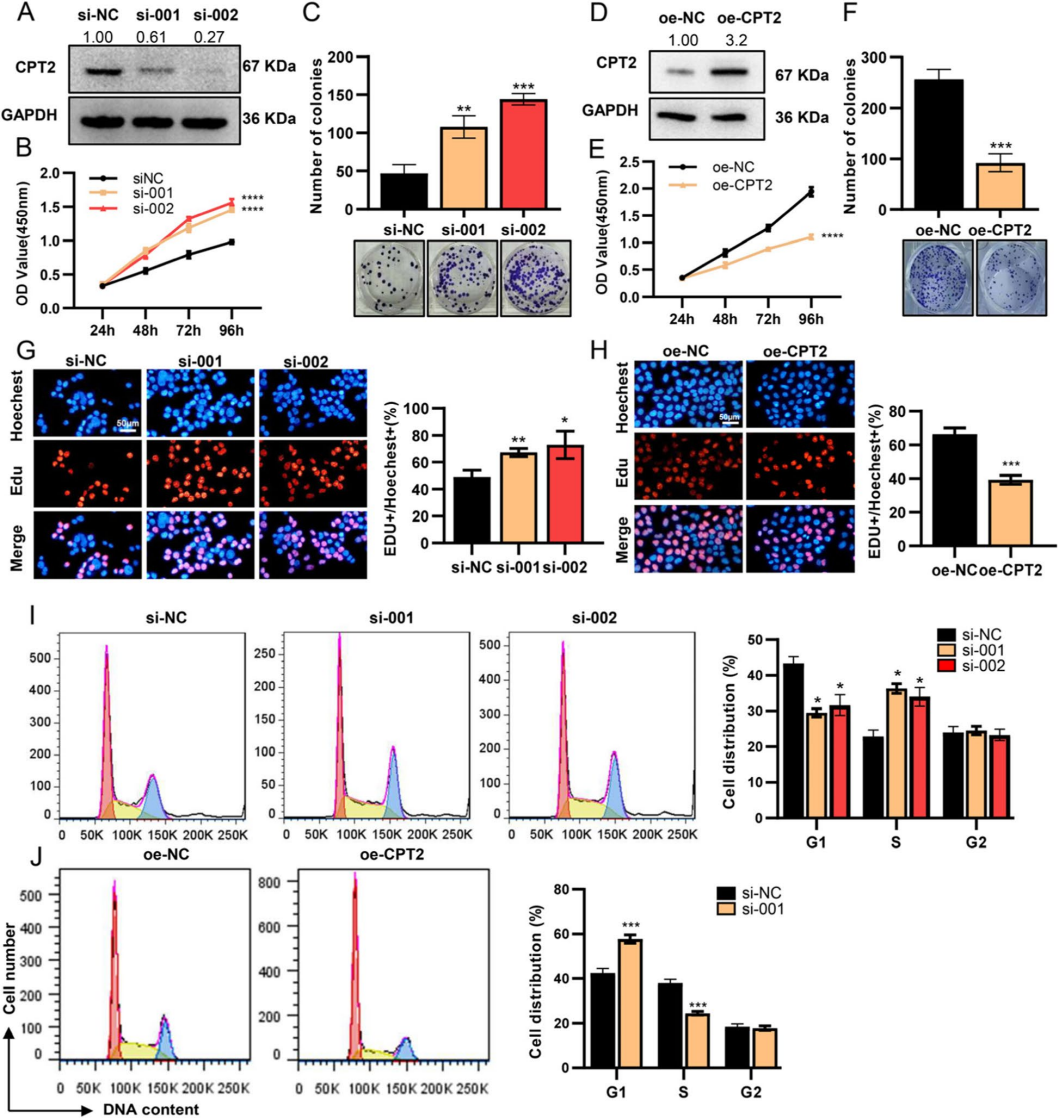
Down-regulated CPT2 promotes cell proliferation in colorectal cancer. **A** The knockdown of CPT2 in HCT116 cells was identified by Western blotting. **B**, **C** The capability of cell proliferation of HCT116 was determined by CCK8 (**B**) and cell clone-formation (**C**) assay. **D** The overexpression of CPT2 in SW620 cells was identified by Western blotting. **E**, **F** The capability of cell proliferation of SW620 was determined by CCK8 (E) and cell clone-formation (**F**) assay. **G**, **H** The capability of cell proliferation of HCT116 (G) and SW620 (**H**) was determined by Edu assay. Scale bar: 50 μm. **I, J** Distribution of cells in three cell cycle phases of HCT116 (I) and SW620 (J) was examined by flow cytometry assay. Data are presented as mean ± SD from three independent experiments. *P < 0.05, **P < 0.01, ***P < 0.001, ****P < 0.0001

In order to investigate the biological role and the underlying mechanism of CPT2 in CRC, we maintained CRC cell lines as following: HCT116 and SW480 cells with relatively higher expression of CPT2 were transfected two different siRNAs (Fig. 6A, Supplementary Fig. 3A), while SW620 with lower expression of CPT2 were transfected with wild-type (WT) full-length CPT2 vectors (Fig. 6D) . CCK-8 assay and colony formation assay were carried out to explore the effect of CPT2 on the growth properties. The analysis showed that the knockdown of CPT2 distinctly promoted colony formation and cell proliferation of HCT116 and SW480 cells (Fig. 6B-C, Supplementary Fig. 3B-C), whereas over-expression of CPT2 significantly suppressed the growth of SW620 cells (Fig. 6E-F). The EdU assay further confirmed the proliferation-suppress effect of CPT2 on CRC cell lines (Fig. 6G-H, Supplementary Fig. 3D).

To figure out how CPT2 affects cell growth, we analyzed the phase distribution of the cell cycle by flow cytometric analysis. It was observed that the knockdown of CPT2 efficiently brought about a decrease of cell percentage in the G1 phase, and an increase of cell percentage in the S phase (Fig. 6I), whereas over-expression of CPT2 caused the inhibition of cell growth by arresting the cell cycle in the G1 phase (Fig. 6J). Overall, these findings come up with evidence that CPT2 could regulate the proliferation of CRC cells in vitro.

## Discussion

Recently, accumulating shreds of evidence have revealed that the therapeutic approaches against the progression of CRC have evolved and the molecular mechanisms of CRC formation and distribution are better understood [25, 26]. However, the patient’s chances of recovery from CRC are still poor. Therefore, there is an urgent need for more evidence about new CRC prediction and prognostic biomarkers.

Typically, cancer cells undergo diverse metabolic rearrangement to meet the needs of cancer phenotype [27]. Emerging evidence reported many metabolic deregulations, like aerobic glycolysis, fuel cell proliferation serine, and glutamine metabolism, fatty acid synthase, survival, and metastasis [28–30]. In the past few years, Warburg theory has been reanalyzed by researchers and have gained a deep understanding of the “metabolic conversion” in cancer cells, which includes the close and causal relationship between cancer genes and metabolic alterations, and their capabilities to be used as targets for the treatment of cancer [27, 31]. Shi et al. found that the metabolic rearrangement induced by EBV-LMP 1 may provide new insights for the treatment and diagnosis of EBV-related cancers [32]. According to Sebastián et al., SIRT6 inhibits the occurrence as well as the progression of CRC via inhibiting aerobic glycolysis and ribosomal gene expression [33]. DeBerardinis et al. reported that the abnormal activation of mTORC1, the loss of tumor suppressor factors (such as p53), and the activation of oncogenes (such as MYC) synergistically induce the metabolic pathways of tumorigenesis [34]. However, there is a lack of clarity, regarding the extensive understanding of these metabolic pathways and their mechanism in the progression of CRC.

In recent decades, the comprehensive bioinformatics analysis of CRC gene expression profile and the development of gene markers associated with the prognosis of CRC have grabbed enough attention of researchers [35]. Ke et al. used the CRC RNA seq data and microRNAs seq data in the TCGA database to construct a CRC ceRNA network to mine the key RNAs that affect the prognosis of CRC [36]. We have also reported that CCNA2 may serve as a new biological marker for diagnosis and assist the combined treatment of CRC through a series of bioinformatics analyses [37]. In the current study, by integrating the metabolism-associated data from the TCGA-CRC and GEO databases, the differentially expressed genes were screened and their prognostic value in CRC was explored. The PPI network of DEGs was established and the three most significant modules and top ten hub genes were filtered. The most significant pathway of module 1 is involved in purine metabolism, pancreatic secretion, and fatty acid degradation. The genes of module 2 are enriched in the metabolism of fatty acid, degradation of fatty acid, and PPAR signaling pathway. The genes of module 3 are significantly enriched in purine metabolism, pyrimidine metabolism and biosynthesis of amino acids. Finally, the survival prognostic analysis and Cox regression analysis were combined to determine CPT2 as an independent factor predicted overall survival.

Abnormal metabolism of lipid was observed as an emerging feature of cancer cells since they bring dysregulations in the expression of genes, proteins, and signaling pathways that are directly or indirectly associated with cancer progression. Given this fact, it can provide a basis for elucidating its pathogenesis and provide potential targets for new and specific cancer therapies [38]. An abnormal fatty acid metabolic activity in cancer progression has gain renewed attention. Luo et al. reported their principal roles in signaling (as secondary messengers), energy storage, act as a fuel source for energy production, and the production of cellular ATP via FAO [39]. In this view, a recent study has evaluated the key role of FAO for the metabolism homeostasis in cancerous cells [14]. CPT2, as a key regulatory enzyme of FAO, plays a vital role in catalyzing the conversion of acyl groups from acylcarnitine to acyl-CoA [13, 15]. According to the latest study, it has been revealed that acylcarnitine accumulation was not only a surrogate marker to downregulate CPT 2 but also directly associated with the occurrence of liver carcinoma [16]. Han et al. revealed the effects of CPT1A/CPT2 and other mitochondrial FAO elements, which can be used as metabolic targets for enhancing the efficacy of radiotherapy for breast cancer [17]. At present times, the expression and function of CPT2 in CRC tissues are still unclear. In the current study, the correlation between CPT2 expression and clinicopathological characteristics of CRC was explored through a series of bioinformatics analyses, and was inferred that the down-regulation of CPT2 expression may be related to the poor prognosis of CRC patients. Moreover, this study revealed that down-regulation of CPT2 expression promotes tumor cell proliferation and provides a new potential target for improving the therapeutic effect of drugs against CRC.

There are several limitations in our study. Firstly, we did not provide direct in vivo evidence for cancer-promoting effects of the CPT2 down-regulation. Therefore, further research is required in advanced in vivo models such as a knockout mouse. Secondly, we did not further explore the mechanism of how CPT2 inhibits the proliferation of colorectal cancer cells, and how it plays a metabolic function in the progression of CRC.

In conclusion, via a series of bioinformatics analyses, the genes that perform metabolic processes in the progression of CRC were extensively investigated. Herein, it was validated that CRC patients with low CPT2 expression tended to have shorter survival time and CPT2 significantly inhibited CRC cell proliferation ability. As a protective prognostic gene, CPT2 might act as a candidate biomarker in the prognostic evaluation of CRC.

## Supplementary Information

**Additional file 1.**

## Data Availability

The TCGA cohort data were available at the Genomic Data Commons (GDC) website (https://portal.gdc.cancer.gov/). Two independent cohorts (GSE44076, GSE21510) of CRC data were downloaded from the GEO database (http://www.ncbi.nlm.nih.gov/geo/). All data needed to evaluate the conclusions in the paper are present in the paper and/or the Supplementary Materials. Additional data are available upon request from corresponding author.
