# Supplementary material for: Identification of CPT2 as a prognostic biomarker by integrating the metabolism-associated gene signature in colorectal cancer
Source: BMC Cancer. 2022 Oct 4;22:1038. doi: 10.1186/s12885-022-10126-0 (PMC9531485; doi:10.1186/s12885-022-10126-0)
Supplement: Supplementary file 1 — Additional file 1. [file 12885_2022_10126_MOESM1_ESM.docx]

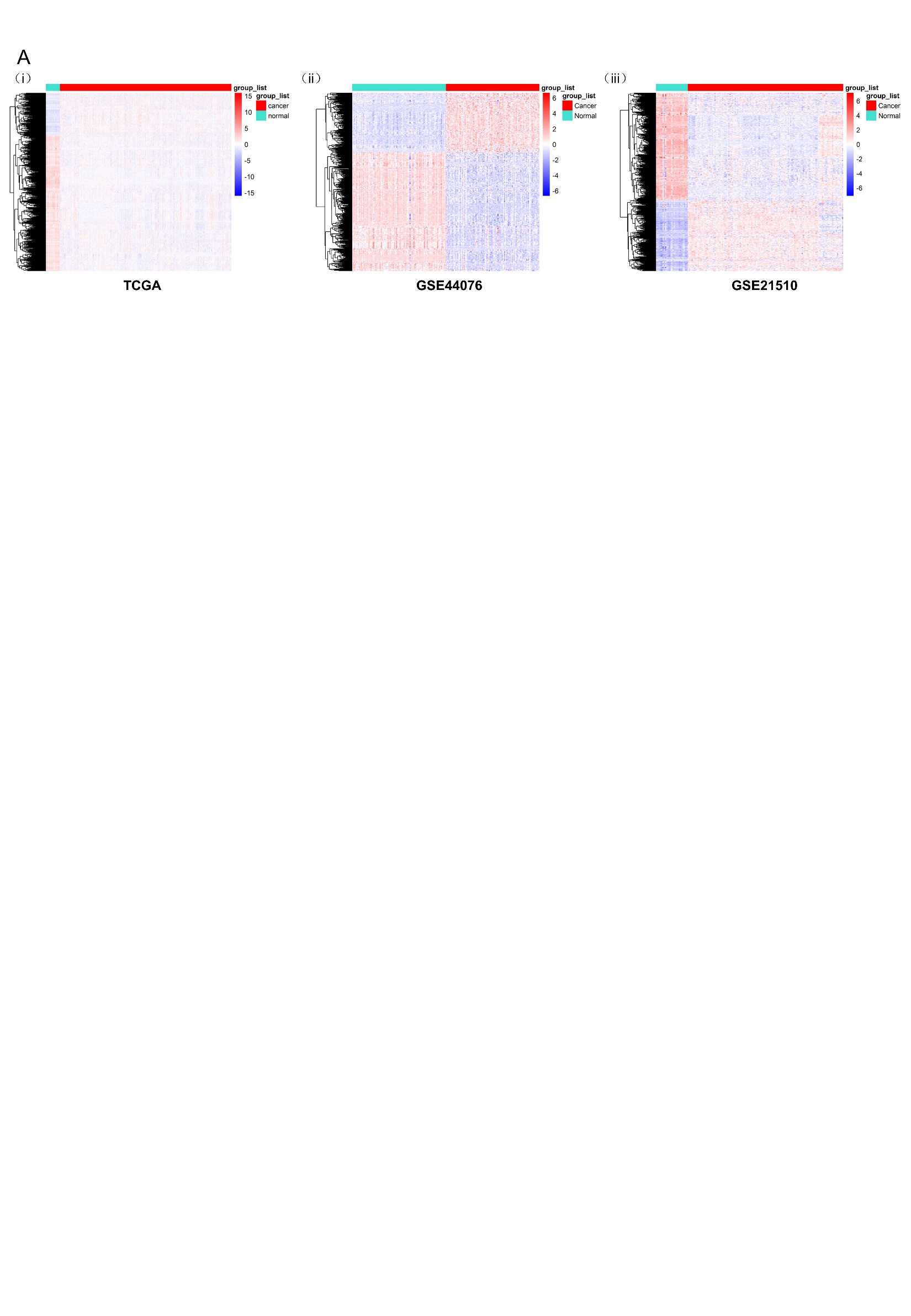


Supplementary Figure 1. The heatmap visualizes the differentially expressed genes in TCGA (i), GSE44076 (ii) and GSE21510 (iii), respectively.


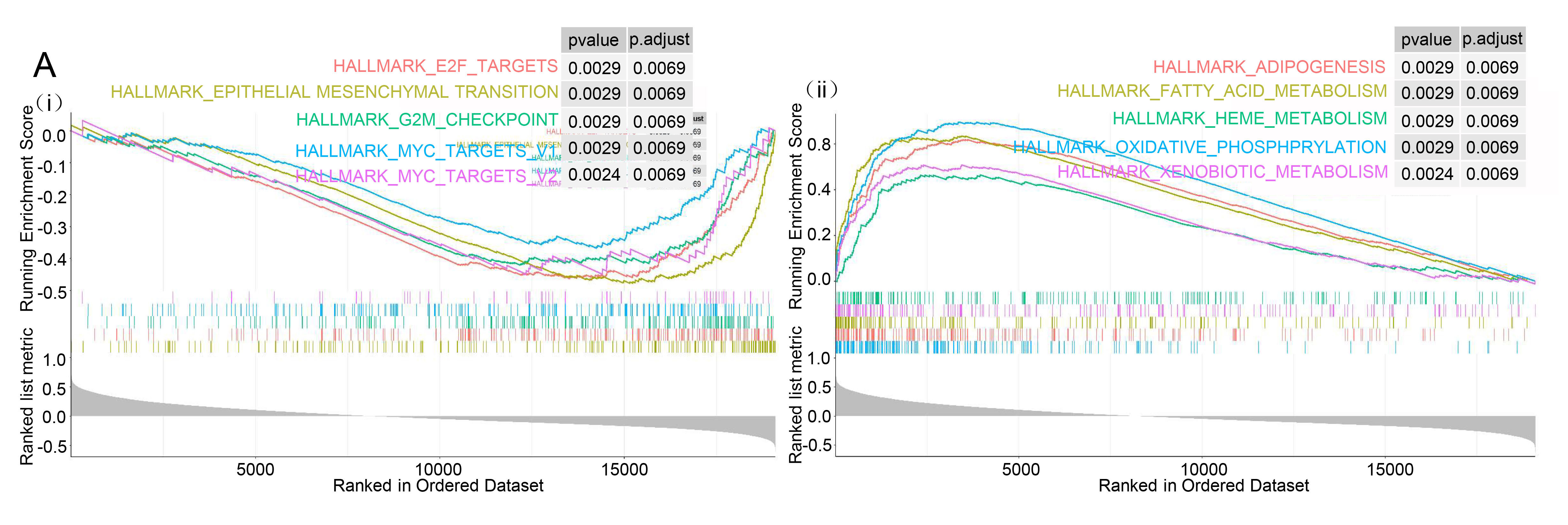


Supplementary Figure 2. The enrichment plot of CPT2 displayed 5 suppressed pathways or activated pathways in (i) and (ii) accordingly.


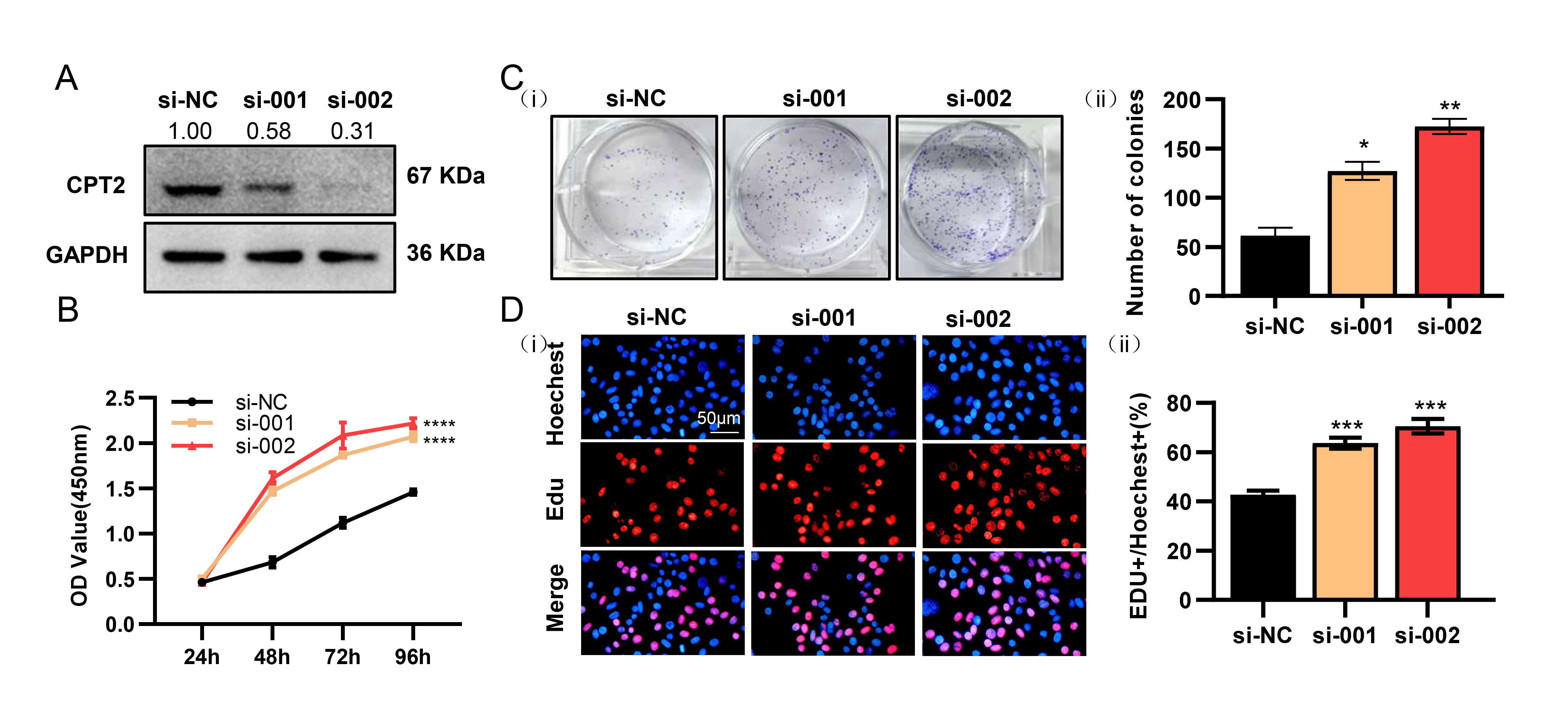


Supplementary Figure 3. Down-regulated CPT2 promotes cell proliferation in colorectal cancer.

(A) The knockdown of CPT2 in SW480 cells was identified by Western blotting.

(B) The capacity of cell proliferation of SW480 was determined by CCK8 assay.

(C) The capacity of cell proliferation of SW480 was determined by cell clone-formation assay, (ii) is the quantification data for (i).

(D) The capacity of cell proliferation of SW480 was determined by Edu assay. (ii) is the quantification data for (i). Scale bar: 50 µm.

Data are presented as mean ± SD from three independent experiments. **P* < 0.05, ***P* < 0.01, ****P* < 0.001, *****P* < 0.0001.


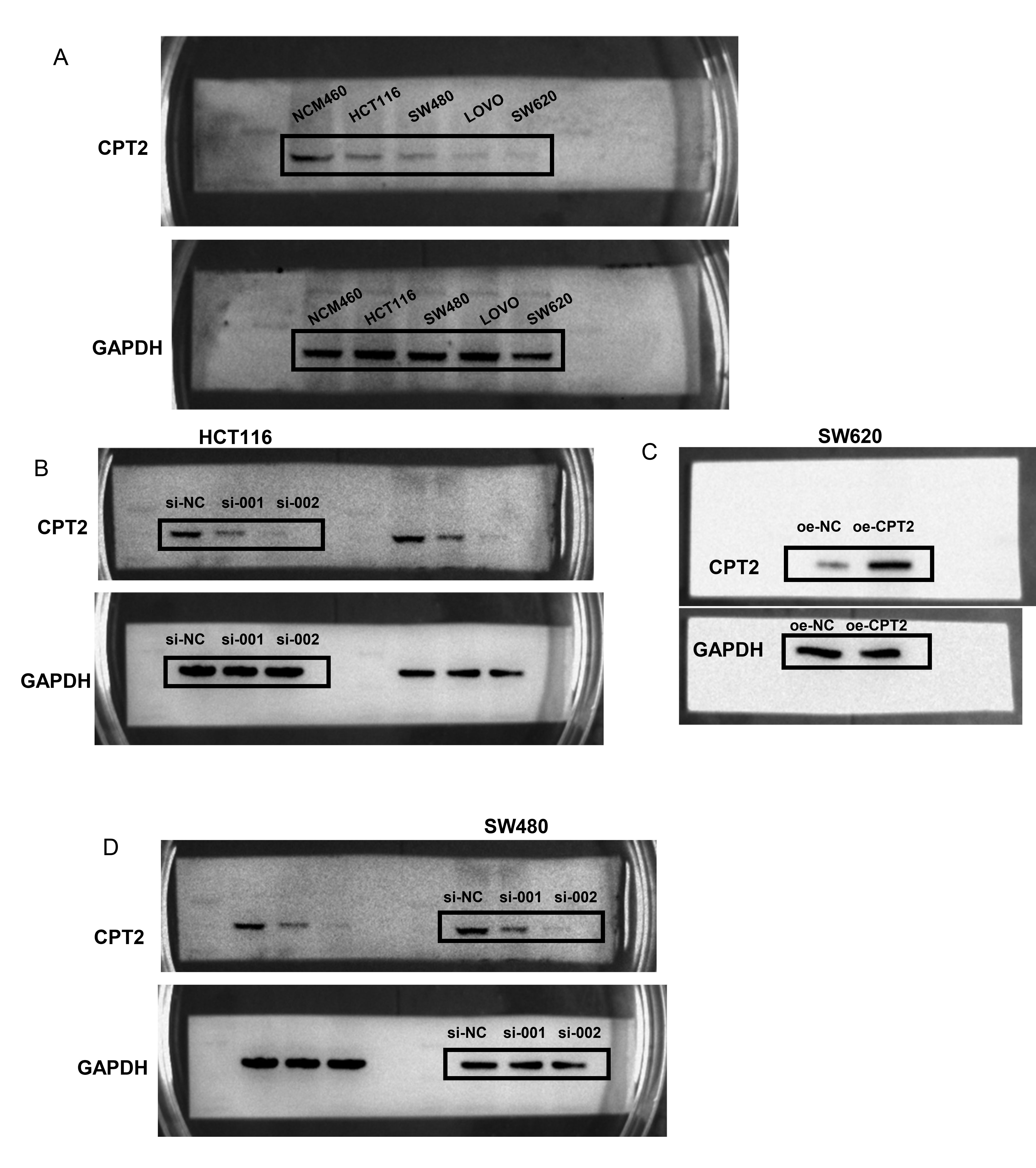


Supplementary Figure 4. The original images of western blot.

(A) Original data of figure 5F.

(B-C) Original data of figure 6A,6D.

(D) Original data of Supplementary Figure 3A.
